# On the Identity and Taxonomic Circumscription of the Pfiesteriacean Genera *Aduncodinium* and *Speroidium* (Dinophyceae)

**DOI:** 10.1111/jeu.70015

**Published:** 2025-06-07

**Authors:** Albert Reñé, Aika Yamaguchi, Takeo Horiguchi, António José Calado, Mona Hoppenrath

**Affiliations:** ^1^ Institut de Ciències del Mar (CSIC), Dpt. Biologia Marina i Oceanografia Barcelona Spain; ^2^ Department of Biological Sciences, Faculty of Science Hokkaido University Sapporo Japan; ^3^ Department of Biology and GeoBioTec Research Unit University of Aveiro Aveiro Portugal; ^4^ Senckenberg am Meer, German Centre for Marine Biodiversity Research (DZMB) Wilhelmshaven Germany

**Keywords:** benthic dinoflagellates, nomenclature, Pfiesteriaceae, phylogeny, taxonomy

## Abstract

With its former circumscription, the genus *Katodinium* included purportedly naked species with a much larger epi‐ than hypocone, several of which were shown to be distantly related to the type species *K. nieuportense*. Most of these species have recently been transferred to other genera, several of them newly established. Among these new genera, *Aduncodinium* was proposed to encompass *Katodinium glandula* (=*Gymnodinium glandula*), and *Speroidium* was erected to encompass *Katodinium fungiforme* (= *Gymnodinium fungiforme* ). However, the genus *Aduncodinium* was invalidly published and several morphological aspects described need to be re‐evaluated. On the other hand, the inclusion in *K. fungiforme* of morphological and molecular characters observed in the strain known as VDH034S “Bullet,” a pfiesteriacean, needs re‐evaluation because the latter shows a higher resemblance to *Aduncodinium* species than to *K. fungiforme*. The present contribution aimed to clarify the identity of the taxa involved on the basis of newly collected morphological and molecular information, and thereby avoid the nomenclatural and taxonomical uncertainties raised by previously published concepts of the genera *Aduncodinium* and *Speroidium*.

## 1 Introduction

The genus *Katodinium* Fott 1957 was erected for nomenclatural reasons to encompass species previously assigned to *Massartia* W.Conrad 1926 because the latter name had previously been used for a fungus (Calado 2011). Species in the genus were characterized by having a remarkably large epicone compared with the hypocone and included several species initially described as members of *Gymnodinium* F.Stein 1878. The type species *K. nieuportense* (W.Conrad) Fott 1957 is thought to be athecate and possess yellowish chloroplasts. However, it has not been observed as its description and thus its characters, generic diagnosis, and phylogenetic affinities remain doubtful (Calado 2011). Many species assigned to *Katodinium* showed no resemblance to the type species and were demonstrated to be thecate and/or showed affinities to other known genera. For instance, *K. rotundatum* (Lohmann) Loeblich III 1965 was transferred to *Heterocapsa* F.Stein 1883, as *H. rotundata* (Lohmann) Gert Hansen 1995), and *K. dorsalisulcum* Hulburt, McLaughlin & Zahl 1960 to *Gymnodinium*, as *G. dorsalisulcum* (Hulburt, J.A.McLaughlin & Zahl) Sh.Murray, de Salas & Hallegraeff 2007 (Murray et al. 2007). Several *Katodinium* species were transferred to the new genus *Opisthoaulax* Calado 2011 based on the presence of tovelliacean characters, such as an eyespot of type C, a large striated root connective attached to the proximal part of the transverse striated root, a tubular pusule with round diverticula with constricted bases, and the production of *Tovellia*‐like cysts (Calado 2011). *Katodinium glaucum* (M.Lebour) Loeblich III 1965 was synonymized with the type of the new genus *Kapelodinium*, *K. vestifici* (F.Schütt) Boutrup, Moestrup & Daugbjerg 2016 (Boutrup et al. 2016).

The genus “*Aduncodinium”* Kang, Jeong & Moestrup 2014 was proposed with *Katodinium glandula* (Herdman) Loeblich III 1965 (=*Gymnodinium glandula* Herdman 1924) as type, although the publication of the name was technically invalid for lack of a full and direct reference to the place of publication of the basionym. The species was originally found in sandy sediments from Port Erin (Irish Sea). The cells were ovoid and flattened, with the episome helmet‐like, the apex produced into a sharp point. The girdle was post‐median without displacement. The hyposome was half the height of the episome and not quite as wide. The sulcus ran obliquely to the left side on the hyposome. The nucleus was spherical and situated in the middle of the body. The cells were colorless and red or yellow bodies were often present. The length of the cells was 20–35 μm (Herdman 1924). The new specimens assigned to “*Aduncodinium glandula* (Herdman) Kang, Jeong & Moestrup 2014” were found in planktonic samples from Korea (Kang et al. 2015). Its plate formula was Po X 4′ 2a 6″ 7c PC 3+s 5″′ 0p 2″″ and molecular information placed the species within the Pfiesteriaceae.

In the same way, the genus *Speroidium* Moestrup & Calado 2018 was lately erected to encompass fungiform *Katodinium* species with an episome longer and wider than the hyposome (Moestrup and Calado 2018) and with *Speroidium fungiforme* (Anisimova) Moestrup & Calado 2018 (=*Katodinium fungiforme* (Anisimova) Loeblich III 1965) as type. The freshwater species *S. austriacum* (J.Schiller) Moestrup & Calado 2018 (=*K. austriacum* (J.Schiller) Loeblich III 1965) was also transferred to this genus, although it is not clear whether it is distinguishable from *S. fungiforme*. The new combination was based on the morphological information available for *Gymnodinium fungiforme* Anisimova 1926 (Anisimova 1926; Spero 1982) and included also molecular information and details of the plate arrangement of clone “Bullet” of strain VDH034S (Seaborn et al. 2006). Moestrup and Calado (2018) stated that the “Bullet” specimens agreed in shape with Anisimova (1926) and Spero (1982) and could be considered to represent *K. fungiforme*. Based on published scanning electron microscopy (SEM) images (Seaborn et al. 2006), the plate formula of “Bullet” was given as 4′? 2a 6″ ?c ?s 5″′ 2″″ and it showed a close phylogenetic relationship with *A. glandula* within Pfiesteriaceae. The morphological and molecular resemblance of specimens shown in Seaborn et al. (2006) with *G. glandula* suggests that both could belong to the same genus.

The objective of this study was to clarify nomenclatural and taxonomical uncertainties surrounding the two genera *Aduncodinium* and *Speroidium*, and the combinations *A. glandula* and *S. fungiforme*. Morphological and molecular information derived from newly collected “*Katodinium*” specimens have been used for this purpose. Available information for other *Katodinium* species, like *K. asymmetricum* (Massart) Loeblich III 1965 , has been included to determine their relationship with *Aduncodinium* representatives.

## 2 Material and Methods

### 2.1 Sampling and Microscopy Observations

Sand samples were collected at Suma Beach, Kobe, Japan (34°38′29″ N, 135°6′48″ E) on May 22, 2013. The sand samples were placed in a plastic Petri dish with Daigo's IMK medium (Nihon Pharmaceutical Co., Tokyo, Japan). This enrichment culture was maintained at 5°C in an incubator under fluorescent light with a 16:8 h light: dark (L:D) cycle. Forty days later, one cell of *Katodinium* Suma was isolated under the inverted microscope by micropipetting into a well of a 24‐well plate containing Daigo's IMK medium and the cells of *Pyrenomonas helgolandi* (SAG28.87) as prey. After the number of cells of *Katodinium* Suma increased, the culture was transferred and maintained in the wells of a 24‐well plate with fresh Daigo's IMK medium and *P. helgolandi*. These culture plates were incubated under the same conditions as the original enrichment culture. Differential interference contrast (DIC) light micrographs and digital videos of live cells were generated using a BX‐50 compound microscope with Nomarski optics (Olympus Optical Co., Tokyo, Japan) equipped with a VB‐7000 digital camera (Keyence, Tokyo, Japan). For fluorescence microscopy, the cultured cells were fixed with 3% paraformaldehyde (final concentration) at room temperature. The fixed cells were put onto a glass slide coated with poly‐L‐Lysin for 10 min and then rinsed with PBS buffer by micropipetting. The fixed cells were treated with 0.1% Sybr‐Green I to stain nuclei and Calcofluor White (Sigma‐Aldrich, UK) to assess thecal plate patterns for 10 min. After washing with PBS buffer, the stained cells were observed with an Olympus BX epifluorescence microscope (Olympus Optical Co., Tokyo, Japan).

An intertidal sandy sediment sample was also collected during low tide in Wilhelmshaven, Germany, at the “Fliegerdeich” site of the south beach (53°30′36″ N, 8°07′43″ E), on September 23, 2013. *Katodinium* cf. *asymmetricum* cells were extracted from the sand with the melting seawater ice method (Hoppenrath et al. 2014; Uhlig 1964) and single cells were isolated by micropipetting using a Leica DMIL inverted microscope (Leica Microsystems GmbH, Wetzlar, Germany). Twenty picked cells were washed twice in dH_2_O and pooled.

The list of the different strains and isolates obtained in this study, as well as information on the origin of different strains and species later discussed, are provided in Table 1. All of them have been circumscribed to the genus *Katodinium*.

**TABLE 1:** List of strains and species related to the genus *Katodinium* discussed, including their geographic origin, diagram included in this study, and bibliographic reference.

Organism	Strain/isolate, origin	Figure	References
*Aduncodinium glandula*	Suma, Japan	4A	**This study**
*Aduncodinium glandula*	Korea	4C	Kang et al. (2015)
*Gymnodinium glandula*	Port Erin, UK	4B	Herdman (1924)
*“Katodinium”* cf. *asymmetricum*	Germany		**This study**
*“Katodinium” asymmetricum*	ExtE, Germany		Reñé et al. (2024)
*Gymnodinium asymmetricum*	Nieuwpoort, Belgium	4E	Massart (1920)
*Speroidium fungiforme*			Moestrup and Calado (2018)
*Gymnodinium fungiforme*	Russia	5A	Anisimova (1926)
Dinophyceae sp. “Bullet”	VDH034S, USA	4D	Seaborn et al. (2006)
*Katodinium nieuportense*	Nieuwpoort, Belgium		Conrad (1926)

### 2.2 DNA Extraction, Amplification, and Sequencing

For the molecular analysis of *Katodinium* Suma, single‐cell isolates, which were transferred to a 24‐well plate with fresh Daigo's IMK medium without prey, were used. After confirmation that prey cells were not present, isolates were, respectively, transferred to 200‐μL PCR tubes containing 10 μL of Quick Extract FFPE DNA Extraction Solution (Epicentre, Madison, WI, USA) and incubated for 1 h at 56°C, then for 2 min at 98°C. For German specimens, genomic DNA was extracted using a MasterPure Complete DNA and RNA Purification Kit (Epicentre, Madison, Wisconsin, USA) according to the manufacturer's protocol. Air‐dried DNA samples were shipped to Hokkaido University, Japan, for molecular analyses. These extracts were used as DNA template for PCR amplification. The initial PCR was performed using a total volume of 25 μL with EconoTaq 2X Master Mix (Lucigen Corp., Middleton, WI) using universal eukaryote primers (SR1: 5′‐TACCTGGTTGATCCTGCCAG‐3′, 25F1: 5′‐CCGCTGAATTTAAGCATAT‐3′ and LSU R2: 5′‐ATTCGGCAGGTGAG TTGTTAC‐3′) (Yamaguchi et al. 2016) for amplifying the SSU (small subunit), ITS1‐5.8S‐ITS2, and the D1–D3 region of LSU (large subunit) rDNA sequences. The PCR protocol had an initial denaturation stage at 94°C for 2 min; 30 cycles of denaturation at 94°C for 30 s, annealing at 52°C for 30 s, and extension at 72°C for 2 min; and final extension at 72°C for 7 min. To obtain the SSU, ITS1‐5.8S‐ITS2, and the D1–D3 region of LSU rDNA sequences, the first PCR product was used as a DNA template for a nested or semi‐nested PCR, where the following combinations of primer pairs were used separately: SR1b and SR3, SR1b and SR5TAK, SR4 and SR7TAK, SR4 and SR9p, SR8p and SR 12, SR10 and SR12b, SR12cF and ITS4_pf_rev (5′‐TCCTCCGCTTACTTATATGC‐3′), 25F1 and 25R1, LSU D3A and LSU R2 (Takano and Horiguchi 2004, 2005; Yamaguchi et al. 2016). The PCR protocol was conducted using an initial denaturation stage at 94°C for 2 min, followed by 25 cycles of 94°C for 30 s, annealing at 50°C for 30 s, and extension at 72°C for 30 s, and final extension at 72°C for 7 min. Amplified DNA fragments corresponding to the expected size were separated by agarose gel electrophoresis and cleaned using the UltraCleanTM 15 DNA Purification Kit (Mo Bio Laboratories, California, USA). The cleaned PCR products were sequenced directly by Fasmac sequencing service (Fasmac, Kanagawa, Japan). The results were confirmed by sequencing both forward and reverse strands and acquired fragments were merged. For *Katodinium* cf. *asymmetricum* Germany, only the SSU rDNA sequences could be obtained. New sequences have been deposited in DDBJ/EMBL/GenBank under the accession number PV292301 (*Aduncodinium glandula*/“*Katodinium*” Suma) and PV292344 (*Katodinium* cf. *asymmetricum* Germany).

### 2.3 Phylogenetic Analysis

Representative molecular sequences of Pfiesteriaceae diversity, including SSU and LSU rDNA genes, were obtained from NCBI to perform phylogenetic reconstructions. The two datasets were aligned using MAFFT v7 under default options, manually curated, and then merged, concatenating the sequences corresponding to the same strain (Table S1). The final concatenated dataset contained 68 sequences and 2704 positions. A maximum likelihood (ML) phylogenetic tree was constructed with RAxML v8.2.12 (Stamatakis 2014), inferring the best tree from 1000 different starting trees and using GTR + GAMMA as the model. Bootstrap statistical support was evaluated using 1000 pseudoreplicates. A Bayesian reconstruction was conducted using MrBayes v3.2.1 (Ronquist et al. 2012), based on the GTR + I + GAMMA model, four MCMC chains, and 1,000,000 generations. Convergence of the MCMC analyses was confirmed in that the average standard deviation of split frequencies was below 0.01, the potential scale reduction factor was close to 1, effective sample sizes were > 200, and no trends were observed in the plots of generations versus log probability. The first 10% of trees were then considered as burned in, and the Bayesian posterior probabilities (BPP) were determined for the majority rule consensus tree. Additional single‐gene phylogenies were constructed for both individual SSU and LSU rDNA datasets following the same procedures previously described. The SSU rDNA alignment contained 54 sequences and 1784 positions, and the LSU rDNA alignment contained 48 sequences and 919 positions.

## 3 Results

### 3.1 Morphological Description of “*Katodinium*” Suma

**FIGURE 1:**
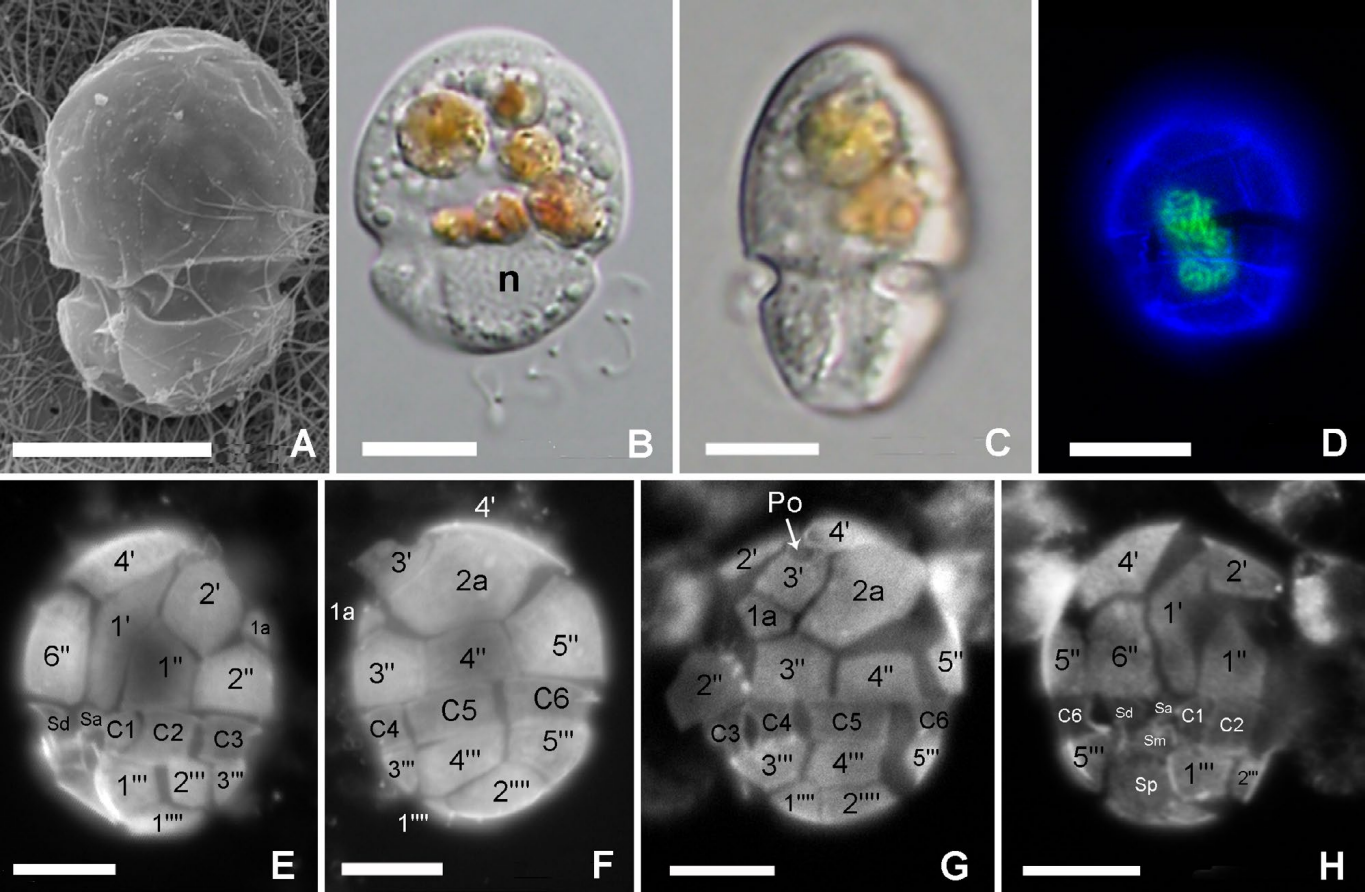
Microscopy images of *Aduncodinium glandula* Suma. (A) Ventral view by SEM. (B) Ventral view by LM. The nucleus can be seen in the left hyposome (n) and numerous colorless granules and colored food particles are present in the episome. (C) Right lateral view showing the dorsoventral compression of the cell. (D) Epifluorescence image of a cell stained with Sybr‐Green showing the elongated nucleus (green) in a central position, probably under division phase. (E–G) Epifluorescence images of Calcofluor white stained cells. (E) Ventral view. (F) Dorsal view, (G) left lateral to dorsal view. (H) Ventral to right lateral view. Scale bars = 10 μm.

Specimens agreeing with the *G. glandula* original description and named as “*Katodinium*” Suma were obtained in coastal sediments from Suma (Japan) in this study and subsequently isolated, cultured, and characterized. The specimens were thecate, with a plate formula APC 4′ 2a 6″ 6c 4s 5″′ 2″″ (Figures 1E–G and 4A). The thecal plates were smooth with pores (Figure 1A). Cells were 11.5–23.5 μm long and somewhat obliquely dorsoventrally flattened (Figure 1B,C). The episome was helmet‐shaped, with a small apical hook bent to the left side of the cell (Figure 1B). The postmedian cingulum showed no displacement (Figure 1A,E). The sulcus was shifted to the right side of the cell and ran slightly obliquely to the left, reaching the antapex (Figure 1A,E). When the cell contained food particles in the episome, a large round nucleus was visible in the left side of the hyposome (Figure 1B). The elongated nucleus, which was sometimes seen to be undergoing division, was located in the middle of the cell when the cell did not contain any food particles (Figure 1D). The cells had no chloroplasts, and numerous granules were observed, mostly located at the episome, as well as yellowish to red ingestion bodies (Figure 1B,C). The cells swam rapidly on the bottom of the culture plates and frequently changed direction. Although cells sometimes stopped swimming and rested on the bottom, they did not strongly attach to the bottom surface.

**FIGURE 2:**
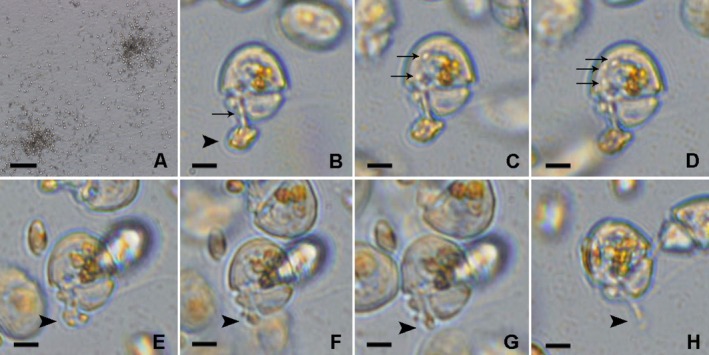
Feeding mechanism of *Aduncodinium glandula* Suma on the cryptophyte *Pyrenomonas helgolandi*. (A) *Aduncodinium* cells swarming around prey cells. (B–H) Single cells showing tube feeding. Arrowheads indicate the prey. Arrows indicate ingestion of particles which penetrate into the dinoflagellate cell through the peduncle (B), and accumulate inside the cytoplasm (C, D). Scale bars = 200 μm (A) and 10 μm (B–H).

The cultured cells fed on cryptomonad cells of *Pyrenomonas helgolandi* by using a peduncle (feeding tube) (Figure 2). The peduncle protruded from the upper part of the sulcus and attached to the prey cell (Figure 2B–D). The cell contents of *P. helgolandi* were drawn up to the episome of *Katodinium* Suma (Figure 2C,D). The cells can swim around during feeding and the prey was consumed in around 30 s (Movie S1). When the food supply was scarce and the cultured cells were food‐deprived (no food particles inside the episome), many *Katodinium* Suma cells were seen swarming around a single prey cell and scrambling to capture it (Figure 2A). It was observed that *Katodinium* Suma was also able to feed on an unidentified diatom and sustain growth; the ingestion process lasted also in the order of 30 s (not shown).

### 3.2 Phylogenetic Relationships

**FIGURE 3:**
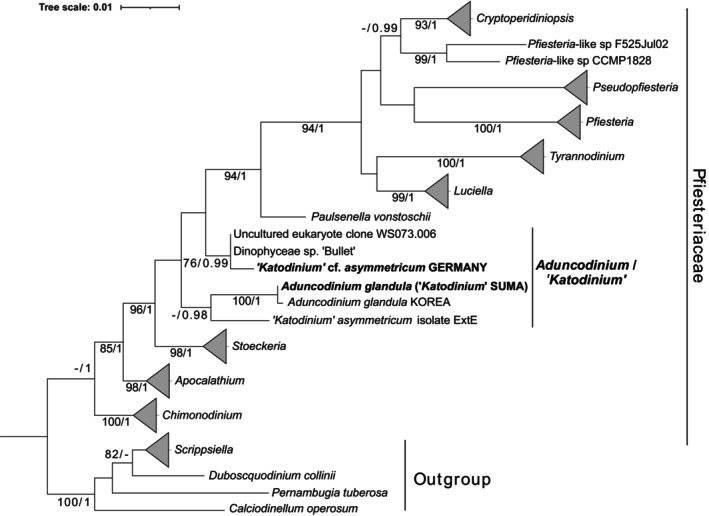
Phylogenetic tree, including concatenated SSU and LSU rDNA sequences of Pfiesteriaceae representatives. Outgroup sequences are represented by Thoracosphaeraceae representatives. Sequences generated in this study are in bold. Values in the nodes represent bootstrap statistical support (%) and Bayesian Posterior Probability. Only values > 70% and > 0.95, respectively, are shown.

The sequences of SSU, 5.8S and LSU rDNA obtained for “*Katodinium”* Suma were almost identical to those corresponding to *Aduncodinium glandula* LK934662 strain from Korea, showing 99.94% identity, with two out of 3165 base pairs (bp) differing positions located in the LSU rDNA gene. This suggests that the strains from Japan (this study) and Korea represent the same species. Phylogenetic analyses using concatenated SSU and LSU rDNA sequences (Figure 3) showed that the sequences of interest are included within the family Pfiesteriaceae. However, these sequences clustered into two clades. The first clade included sequences of *A. glandula* and “*Katodinium”* Suma, and *K. asymmetricum* isolate ExtE from Germany. The second one included *K*. cf. *asymmetricum* Germany, Dinophyceae sp. “Bullet” and an environmental sequence. In both cases, their bootstrap statistic support was low, but BPP was consistently robust (0.99 and 0.98, respectively). The first clade was also recovered in the constructed LSU rDNA phylogeny (Figure S1), in this case showing high statistical support (99% bootstrap/1 BPP). However, LSU rDNA sequences representing the second clade are not available. Regarding the constructed SSU rDNA phylogeny (Figure S2), *A. glandula* and “*Katodinium”* Suma sequences formed a sister clade to a cluster including *K*. cf. *asymmetricum* Germany, Dinophyceae sp. “Bullet,” and an environmental sequence (84%/1). However, the relationship between the two clades showed no statistical support and *K. asymmetricum* ExtE clustered independently of the other sequences of interest.

The complete SSU rDNA of “*Katodinium”* cf. *asymmetricum* Germany showed 99.7% identity to sequence AY251288 Dinophyceae sp. “Bullet” strain VDH034S, with 6 of 1737 bp differing positions. The SSU rDNA environmental sequence KP404754 obtained by cloning from coastal South China Sea samples was identical to the VDH034S strain sequence. Finally, sequence AY251288 showed 98.9% identity to the SSU rDNA sequence of *A. glandula* LK934662, with 11 of 1739 bp differing positions. Unfortunately, the LSU rDNA sequence of strain VDH034S is missing and that of *K*. cf. *asymmetricum* Germany could not be obtained. As previously shown, those sequences clustered independently to *Aduncodinium glandula* and “*Katodinium”* Suma in the concatenated phylogeny (Figure 3), but forming sister clades in SSU rDNA phylogeny (Figure S2), even though showing no statistical support. Consequently, the interpretation that they belong to the same genus must be treated with caution, at least until LSU rDNA sequences are obtained for all representatives, given their higher taxonomic robustness compared with SSU rDNA.

## 4 Discussion

### 4.1 Correspondence Between *Katodinium* Suma and *Gymnodinium glandula*

**FIGURE 4:**
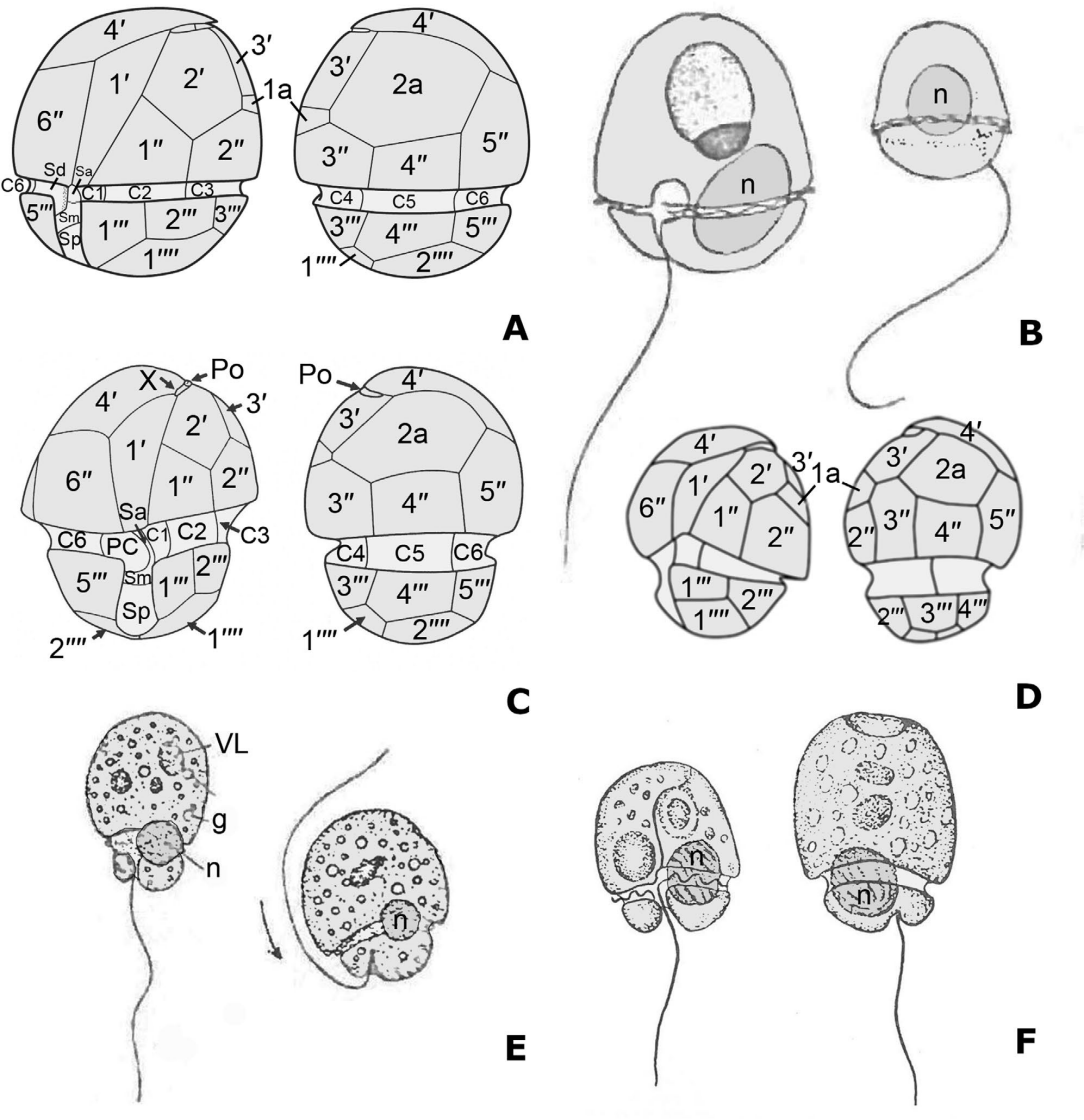
Ventral and dorsal schematic drawings. (A) *Aduncodinium glandula* (this study). (B) *Gymnodinium glandula* modified from Herdman (1924). (C) *Aduncodinium glandula* modified from Kang et al. (2015). (D) Dinophyceae sp. “Bullet” strain VDH034S modified from Seaborn et al. (2006). (E) *Gymnodinium asymmetricum* modified from Massart (1920). (F) *Massartia asymetrica* modified from Biecheler (1952).

The first aspect that requires our attention is the correspondence between *Gymnodinium glandula* and the specimens studied by Kang et al. (2015), from which DNA was extracted and used to infer the phylogenetic affinities of *Aduncodinium glandula*. *Gymnodinium glandula* was described by E.C. Herdman in 1924 from coastal sediments obtained at Port Erin, Isle of Man. The benthic species was described as naked, having flattened ovoid cells, with a helmet‐shaped epicone and a sharp point bent backwards at the apex (Figure 4B). The cingulum was postmedian without displacement. The hypocone was half the height of the epicone and slightly narrower. The sulcus ran obliquely to the left on the hypocone and had a short extension that penetrated the epicone. The nucleus was described as spherical and situated in the middle of the cell, even though it is clearly located in the lower left side of the cell and partly extends into the epicone in Herdman's (1924) figure 30. The cells were colorless and showed refractile granules. A red body was often present in the epicone and a round pusule was depicted in Herdman's (1924) figure 30. The cells were 20–35 μm long. Other than the fact that *G. glandula* was not initially recognized as thecate, all characteristic features of the specimens studied herein are in agreement with the original description, supporting the interpretation that they belong to that species.

### 4.2 Validation of *Aduncodinium glandula*

**FIGURE 5:**
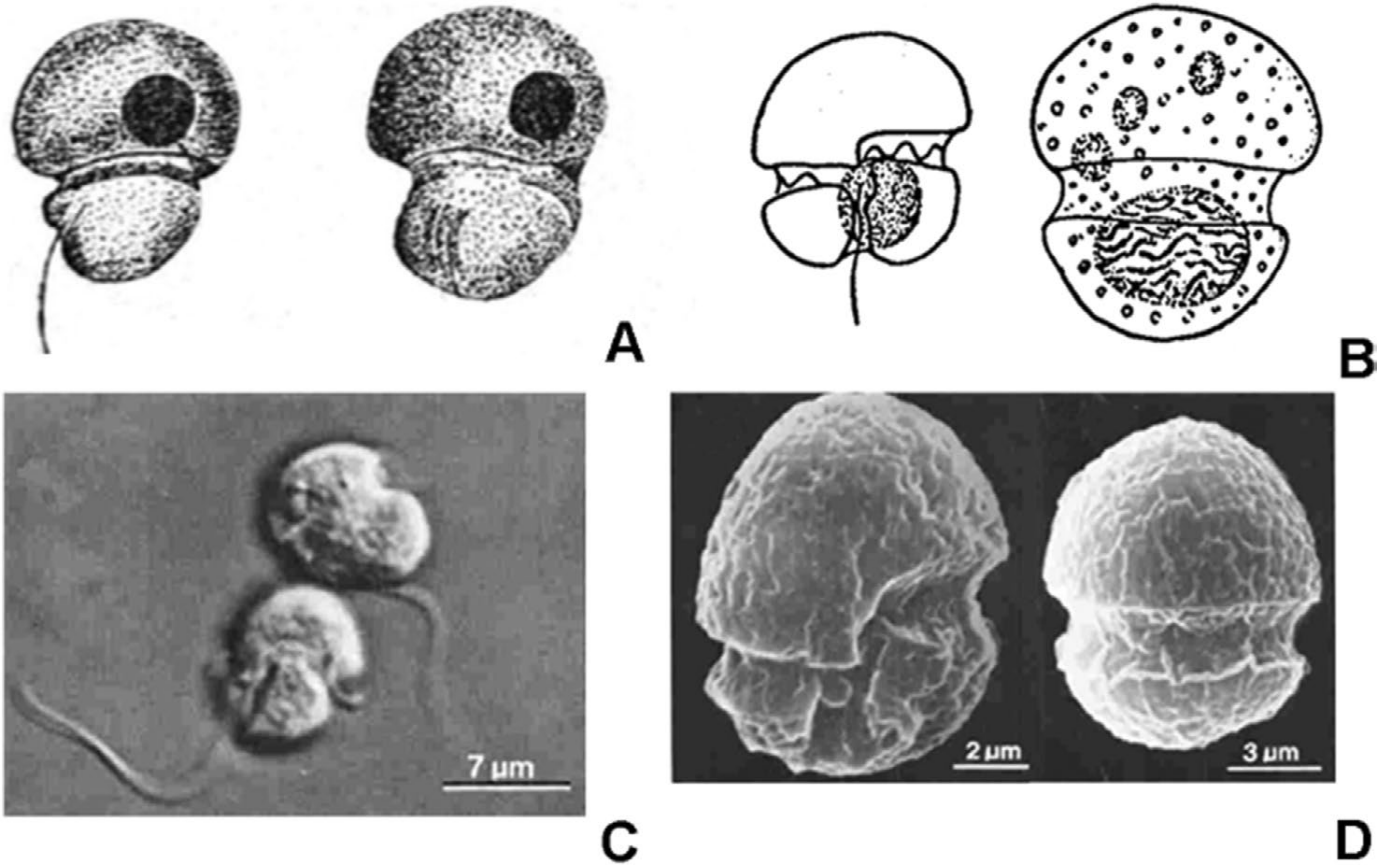
Images of *Speroidium fungiforme* (= *Gymnodinium fungiforme* ). (A) Drawings from Anisimova (1926). (B) Drawings from Biecheler (1952). (C) Light microscopy images from Spero and Morée (1981). (D) Scanning electron microscopy images from Spero and Morée (1981).

The specimens studied from Suma were benthic, whereas those described by Kang et al. (2015) were found in plankton samples. However, comparison between DNA sequences of the specimens studied by Kang et al. (2015) and the ones studied herein suggests in turn that they are conspecific. They also show high morphological agreement and we concur that the specimens shown by Kang et al. (2015) correspond to *Gymnodinium glandula*. Unfortunately, the genus *Aduncodinium* was invalidly published (Art. 41.1 of the International Code of Nomenclature for algae, fungi and plants; Turland et al. 2018) for lack of a full and direct reference to the basionym of the combination *Aduncodinium glandula*, thereby depriving the genus of a designated type (Kang et al. 2015). Although the name *A. glandula* is repeatedly indicated as a new combination in several subtitles within the sections on Materials and Methods and Results, and again when designating the type species of the new genus, the matter is further confused by the addition of the abbreviation “n. sp.” after “comb. nov.” on p. 34, and the designation of a holotype from Masan Bay Korea (Kang et al. 2015). The intention of assigning both the population from Korea and *Katodinium glandula* to a “new genus and a new combination” (Kang et al. 2015, abstract and page 27) is incongruent with the reference to “a new heterotrophic dinoflagellate genus and species” at the beginning of the Discussion (Kang et al. 2015, page 33). In addition, several morphological aspects from the species description in Kang et al. (2015) need to be discussed. As clearly seen in their Figures 2C and 3A, plate 1a is located between 2″ and 3′. However, it has not been depicted in their schematic diagram shown in Figure 5A (reproduced herein as Figure 4C). The authors state that a closing plate (Pi) is absent. However, the APC is covered by the apical hook, and the TEM longitudinal section needs to include the apical pore for that feature to be observed. The absence of a Pi plate is not clearly shown in the published images and cannot therefore be confirmed with the information provided; it is not possible to ascertain whether a Pi plate is present or absent until additional TEM data are available. Finally, the Kofoidian plate formula given in the diagnosis states the presence of seven cingular plates and a peduncle cover plate (PC). In contrast, only six cingular plates are shown in all images provided and mentioned in the plate formula in table 3. The presence of 6c plus PC plates is in agreement with observations done for “*Katodinium”* Suma, even though PC plate is interpreted as a sulcal plate in this study. Therefore, the published plate formula stated in the diagnosis of the genus does not agree with the information provided in Kang et al. (2015). In addition, the genus diagnosis reads “Apical pore plate points to the left cell side.” The Po plate is shifted to the left lateral side but is not “pointing.” If it is the apical hook, which is otherwise not mentioned in the diagnosis, that is meant, then it corresponds to the fourth apical plate. The sulcus shift to the right side of the cell is not described in the genus diagnosis and it is not shown in the line drawing of the species but it is characteristic, in our opinion. Given the discrepancies regarding the formal recombination of *Gymnodinium glandula* to *Aduncodinium glandula*, and the invalidity of the new genus, a formal validation of the genus *Aduncodinium* and a new corrected genus diagnosis is provided.

Formal validation of the genus:

*Aduncodinium* N.S.Kang, H.J.Jeong et Moestrup *ex* A.Yamaguchi et Hoppenrath n. g.

Type: *Aduncodinium glandula* (Herdman) N.S.Kang, H.J.Jeong et Moestrup *ex* A.Yamaguchi et Hoppenrath n. comb.

Description: Thecate dinoflagellate with obliquely dorsoventrally flattened cells. Epicone larger than hypocone. Tabulation (Kofoid system) APC 4ʹ 2a 6″ 6c 4s 5″ʹ 0p 2″″. Cingulum postmedian without displacement or very slightly descending, less than one cingular width. Sulcus shifted to the right cell side, reaching the antapex. Apical hook pointing to the left. APC shifted to the left cell side. Currently known species are heterotrophic, feeding with a peduncle.

Zoobank ID: LSID urn:lsid:zoobank.org:act:E77572FF‐CEce09‐4B43‐902B‐6481054D4B29.

*Aduncodinium glandula* (Herdman) N.S.Kang, H.J.Jeong et Moestrup *ex* A.Yamaguchi et Hoppenrath n. comb.

Basionym: *Gymnodinium glandula* Herdman 1924, *Proceedings and Transactions of the Liverpool Biological Society* 38, page 81, figures 30, 31.

Homotypic synonyms: *Massartia glandula* (Herdman) J.Schiller 1933, 435; *Katodinium glandula* (Herdman) Loeblich III 1965, page 16.

Zoobank ID: LSID urn:lsid:zoobank.org:act:840EA6FA‐AD2D‐419F‐AFF7‐7210939B292C.

### 4.3 Identity of Strain ODU034/VDH034S “Bullet”

Seaborn et al. (2006) published a study providing the molecular information and SEM images of several pfiesteriacean cultures, mostly established from samples taken from the Virginia tidal estuaries of Chesapeake Bay (USA). The specimens of the strain named ODU034/VDH034 “Bullet” were described as having two anterior intercalary plates, which were not in contact, plate 2a being pentagonal. The APC did not have a canal plate (X) and a closing plate was not observed (Figure 4D). Although not described, an apical hook may be clearly seen in their Figure 1E,F. The plate formula given was APC 4′ 2a 6″″ c? s 5″′2″″. All these features were clearly different to the other studied strains, later assigned to new genera like *Luciella* Mason, Jeon, Litaker, Reece & Steidinger 2007 or *Cryptoperidiniopsis* Steidinger, Landsberg, Mason, Vogelbein, Tester & Litaker 2006 (Mason et al. 2007; Steidinger et al. 2006). Cryptoperidiniopsoids have five apical and no anterior intercalary plates, while the APC of *Luciella* species is morphologically distinct, and the 2a plate is quadrangular (Calado et al. 2009). Finally, the “Bullet” strain showed a reduced or absent swarming response to prey presence, in contrast to cryptoperidiniopsoids or *Pfiesteria* Steidinger & J.M.Burkholder 1996 species. All those morphological and behavioral differences were supported by their phylogenetic information, corresponding to the SSU rDNA sequence AY251288, which clustered independently to those corresponding to previously cited genera (Figure 3). The morphological features of the “Bullet” strain are in agreement with the diagnosis of the genus *Aduncodinium*.

More details are needed for a complete characterization of *K*. cf. *asymmetricum* specimens obtained from Germany, but their overall morphology strongly resembles the organism shown by Seaborn et al. (2006), which in turn showed evident similarities to *Gymnodinium asymmetricum* Massart 1920. The species was described by Massart (1920), but a morphological characterization was not given (Figure 4E). Later on, Biecheler (1952) provided a more detailed description of “*Massartia asymetrica*” (Massart) J.Schiller 1933 (=*G. asymmetricum*), but failed to recognize it as a thecate species (Figure 4F). There are numerous references to this species in the literature (see Hoppenrath et al. 2023), including images of their thecal plates, for example, Al‐Qassab et al. (2002); Al‐Yamani and Saburova (2010); Hoppenrath (2000); Hoppenrath et al. (2023); Larsen (1985); Larsen and Patterson (1990). However, a detailed characterization of its plate pattern or molecular information has never been provided. At this stage, it cannot be confirmed that all the specimens observed in the light microscope belong to *Katodinium asymmetricum* . A first examination by SEM of a field sample from Germany revealed morphological differences of specimens that would have been identifiable as *K. asymmetricum* in the LM, so that the taxon most likely represents a species complex of semi‐cryptic species (Hoppenrath et al. 2023). It will be difficult or impossible to decide which of the morphotypes represents the true *K. asymmetricum* unless it is re‐discovered and investigated at the type locality, in Nieuwpoort, Belgium. But all evidence suggests that this species and closely related ones are members of *Aduncodinium*, together with *G. glandula* (=*K. glandula*).

As previously mentioned, Moestrup and Calado (2018) tentatively included features of the strain designated “Bullet” by Seaborn et al. (2006) in their concept of *Speroidium fungiforme* because of the resemblance in external morphology of cells of that pfiesteriacean strain to both the original description of the species by Anisimova (1926) and to the populations studied by Spero (1982). The hypothetic identity between “Bullet” and *S. fungiforme* provided an otherwise unknown tabulation for the species and two drawings showing thecal plates visible in SEM micrographs in Seaborn et al. (2006) were added to a reproduction of Anisimova's (1926) original drawings of *Gymnodinium fungiforme* , while a word of caution was given that more than one species might be involved (Moestrup and Calado 2018, 295). Based on the evidence presented herein, we now argue that “Bullet” is not conspecific with *S. fungiforme*, but represents instead a species of *Aduncodinium*.

### 4.4 Identity of *Gymnodinium fungiforme*

*Gymnodinium fungiforme* was first detected in sediments and in films floating on the surface from a salty lake in Russia. It was described as a very rare organism, 11 μm long, 8.8 μm wide, with the epicone longer than the hypocone, and easily recognizable by its shape and characteristic movement (Figure 5A). The cingulum formed a depression between the larger epicone and the smaller hypocone, resulting in a mushroom shape, justifying the specific epithet “fungiforme.” The sulcus ran somewhat obliquely. The organism commonly showed a dark brown body interpreted as a lipid vacuole. The movement was fast, making stops of different duration, when cells rotated rapidly. Further details mostly regarding the sulcal area and cingulum displacement, but also describing its striking swimming behavior, were provided by Biecheler (1952), based on observations from Thau lagoon (France), in the Mediterranean Sea (Figure 5B). Discrepancies with the original description were provided in relation to the nucleus position, which was placed in the hypocone, while the nucleus, or perhaps the round body that may correspond to an ingestion body, was originally drawn in the epicone. The cells were 10–12 μm long and 8–10 μm wide. As occurred by many other *Gymnodinium* species, it was transferred to the genus *Katodinium*. Studies dealing with *K. fungiforme* are scarce, but some morphological details can be found. Cells have been described as phagotrophic, lacking chloroplasts (Figure 5C), with a left‐handed descending cingulum (Figure 5D). The nucleus occupies the hypocone and food bodies are commonly present (Spero and Morée 1981). A complex life cycle has been described, including asexual vegetative cells, zoosporangia or resting cysts, and sexual gametes and zygotes. Phagotrophic cells are attracted by the presence of prey, forming rapidly moving aggregations, and attaching to the prey and ingesting the cytoplasm through a highly extensible peduncle (Spero and Morée 1981).

In contrast to the presence of an apical hook bent to the left in *G. glandula* and *G. asymmetricum*, an apical hook was never described in *Gymnodinium fungiforme* (Anisimova 1926; Hoppenrath et al. 2014; Spero 1982; Spero and Morée 1981), which speaks against its affinity with *Aduncodinium*. In the case of “Bullet” strain, its swimming behavior was little active (Seaborn et al. 2006) and did not display the characteristics described for *K. fungiforme*. The SEM image of *K. fungiforme* in Hoppenrath et al. (2014) cited by Moestrup and Calado (2018) does not correspond to the organism shown by Seaborn et al. (2006) and neither do the illustrations of the species in Spero (1982) and Spero and Morée (1981) (Figure 5D). Consequently, we argue that characters derived from the strain “Bullet” must be excluded from the description of the genus *Speroidium*, and that a more complete assessment of the features of *S. fungiforme* must await further examination. Below we provide a modified description of the genus *Speroidium* based mainly on information from the original description of *Gymnodinium fungiforme* (Anisimova 1926), its type species, and incorporating matching information from later descriptions of species, including those by Spero and Morée (1981) and Spero (1982), whose detailed descriptions of the pfiesteriacean behavior of the species are quite compatible with the original descriptions of morphology and swimming behavior (see Calado and Moestrup 1997).

*Speroidium* Moestrup et Calado 2018.

Cells small, mostly 9–19 μm long, with episome longer and wider than hyposome, giving them a mushroom‐like appearance. Cingulum descending up to about one cingulum width. Chloroplasts and stigma absent. Phagotrophic species. Ingested food collected in episome, nucleus filling up most of the hyposome. Feeding behavior involving attraction to injured prey through bursts of rapid swimming in a helical pattern alternating with periods of quick rotation in one place. Thecal tabulation pattern so far uncertain. In salt lakes and brackish to marine, coastal water (reports in fresh water need to be confirmed).

It may be noted that the characteristics and available information on *Speroidium fungiforme* do not exclude the possibility that it may be identical to a species of some previously described genus of Pfiesteriaceae, such as *Luciella* or *Cryptoperidiniopsis* (Mason et al. 2007; Seaborn et al. 2006; Steidinger et al. 2006). If that proves to be the case, *Speroidium* must be regarded as a junior synonym of that genus; on the other hand, the epithet *fungiformis*, *‐e* will then have priority, requiring the establishment of a new combination with the generic name to be retained.

## Conflicts of Interest

The authors declare no conflicts of interest.

## Supporting information


**MOVIE S1.** Video recorded by light microscopy showing *Aduncodinium glandula* feeding on the cryptomonad *Pyrenomonas helgolandi* by using a peduncle (feeding tube).

Appendix S1.

## Data Availability

The data that support the findings of this study are openly available in NCBI at https://www.ncbi.nlm.nih.gov.
