# Supplementary material for: On the Identity and Taxonomic Circumscription of the Pfiesteriacean Genera Aduncodinium and Speroidium (Dinophyceae)
Source: J Eukaryot Microbiol. 2025 Jun 7;72(4):e70015. doi: 10.1111/jeu.70015 (PMC12144677; doi:10.1111/jeu.70015)
Supplement: Supplementary file 2 — Appendix S1. [file JEU-72-e70015-s002.docx]

**SUPPLEMENTARY MATERIAL**

**TABLE S1:** List and NCBI accession numbers of sequences used for the reconstruction of SSU, LSU and concatenated rDNA phylogenetic trees. Sequences in bold are obtained in this study.

| **Sequence name** | **Genus** | **SSU rDNA** | **LSU rDNA** |
| --- | --- | --- | --- |
| ***Aduncodinium glandula* SUMA** | ***Aduncodinium*** | **PV292301** | **PV292301** |
| ***‘Katodinium*' cf. *asymmetricum* GERMANY** | ***‘Katodinium’*** | **PV292344** |  |
| *Aduncodinium glandula* | *Aduncodinium* | LK934662 | LK934662 |
| *Katodinium asymmetricum* isolate ExtE | *‘Katodinium’* | ON015028 | ON015071 |
| Uncultured eukaryote clone WS073 | *‘Katodinium’* | KP404754 |  |
| Dinophyceae sp. Bullet | *‘Katodinium’* | AY251288 |  |
| *Apocalathium aciculiferum* strain PABR3 | *Apocalathium* | EF417313 |  |
| *Apocalathium baicalense* isolate BAIK2011 | *Apocalathium* | KF446624 |  |
| *Apocalathium euryceps* isolate ERK2012 | *Apocalathium* | KF446622 |  |
| *Peridinium aciculiferum* | *Apocalathium* |  | KJ450990 |
| *Peridinium aciculiferum* | *Apocalathium* | AY970653 | HQ176321 |
| *Peridinium baicalense* | *Apocalathium* |  | KJ450986 |
| *Chimonodinium* cf. *lomnickii* isolate D916uni | *Chimonodinium* | MW784601 | MW784601 |
| *Chimonodinium* cf. *lomnickii* strain GeoM*826 | *Chimonodinium* | MZ475040 | MZ475040 |
| *Chimonodinium lomnickii* var. *wierzejskii* | *Chimonodinium* | KF446619 | MW267276 |
| *Chimonodinium lomnickii* var. *wierzejskii* strain GeoM*715 | *Chimonodinium* | MH497027 | MH497026 |
| *Chimonodinium lomnickii* var. *wierzejskii* strain GeoM*987 | *Chimonodinium* | MZ475047 | MZ475047 |
| *Peridinium wierzejskii* | *Chimonodinium* | AY443018 |  |
| *Cryptoperidiniopsis brodyi* | *Cryptoperidiniopsis* | AF080097 | DQ991379 |
| *Cryptoperidiniopsis brodyi* strain CBDE14 | *Cryptoperidiniopsis* | DQ991375 | DQ991375 |
| Cryptoperidiniopsoid sp. Folly C5 | *Cryptoperidiniopsis* | AY590481 | AY590481 |
| Cryptoperidiniopsoid sp. H V14 | *Cryptoperidiniopsis* | AY245690 | AY245690 |
| Cryptoperidiniopsoid sp. NOAA Beach | *Cryptoperidiniopsis* | AY590486 | AY590486 |
| Cryptoperidiniopsoid sp. PLO21 | *Cryptoperidiniopsis* | AY245691 | AY245691 |
| *Pfiesteria*-like sp. CCMP1827 | *Cryptoperidiniopsis* | AY456118 |  |
| Uncultured Pfiesteriaceae clone DDI96 | *Cryptoperidiniopsis* | MK177614 |  |
| *Luciella masanensis* clone c1075 | *Luciella* |  | MK236554 |
| *Luciella masanensis* clone c1599 | *Luciella* |  | MK236556 |
| *Luciella masanensis* clone c720 | *Luciella* |  | MK236555 |
| *Pfiesteria*-like dinoflagellate | *Luciella* | AY033487 |  |
| *Pfiesteria*-like dinoflagellate Lucy | *Luciella* | AY245689 | AY245689 |
| *Pfiesteria*-like dinoflagellate strain Jeju Lucy-200504 | *Luciella* | AM050345 |  |
| *Pfiesteria*-like dinoflagellate strain Masan Lucy-200505 | *Luciella* | AM050344 |  |
| *Pfiesteria*-like sp. CCMP1835 | *Luciella* | AY590477 | AY590477 |
| *Pfiesteria*-like sp. HR1NovC5 | *Luciella* | AY590482 | AY590482 |
| *Pfiesteria*-like sp. HR1SSeptA5 | *Luciella* | AY590483 | AY590483 |
| *Pfiesteria*-like sp. NC Lucy-V27 | *Luciella* | AY590485 | AY590485 |
| Pfiesteriaceae sp. *masanensis* isolate VIMS 1041 | *Luciella* | EU048552 | EU048552 |
| Pfiesteriaceae sp. *masanensis* isolate VIMS 1050 | *Luciella* | EU048553 | EU048553 |
| *Paulsenella vonstoschii* | *Paulsenella* | AJ968729 |  |
| *Pfiesteria piscicida* | *Pfiesteria* | AY245693 | AY245693 |
| *Pfiesteria piscicida* | *Pfiesteria* | AF077055 | AY112746 |
| *Pfiesteria piscicida* clone Ppi+Rhod | *Pfiesteria* | FJ600088 | FJ600087 |
| *Pfiesteria piscicida* isolate CCMP 1831 | *Pfiesteria* | AF330620 |  |
| *Pfiesteria shumwayae* | *Pseudopfiesteria* | AY245694 | AY245694 |
| *Pfiesteria* sp. B112456 | *Pseudopfiesteria* | AF218805 |  |
| *Pseudopfiesteria shumwayae* | *Pseudopfiesteria* | AF080098 |  |
| Dinophyceae sp. Shepherds Crook | *Stoeckeria* | AY590479 | AY590479 |
| Dinophyceae sp. Shepherds Crook | *Stoeckeria* | AY590484 | AY590484 |
| *Stoeckeria algicida* | *Stoeckeria* | AJ841809 |  |
| *Stoeckeria algicida* strain SAMS | *Stoeckeria* | HG005134 | HG005133 |
| *Stoeckeria* sp. SSMS0806 | *Stoeckeria* | FN557541 | FN557541 |
| *Stoeckeria* sp. SSSC09 | *Stoeckeria* | HG005132 | HG005132 |
| *Peridiniopsis berolinense* voucher HBI HB201010a | *Tyrannodinium* |  | JQ639755 |
| *Tyrannodinium berolinense* | *Tyrannodinium* |  | FJ167681 |
| *Pfiesteria*-like sp. CCMP1828 | *-* | AY590476 | AY590476 |
| *Pfiesteria*-like sp. F525Jul02 | *-* | AY590480 | AY590480 |
| *Calciodinellum operosum* | Outgroup |  | KF751922 |
| *Duboscquodinium collinii* isolate VSM11 | Outgroup | HM483399 | HM483399 |
| *Pernambugia tuberosa* | Outgroup |  | JN982372 |
| *Scrippsiella acuminata* | Outgroup |  | JN982383 |
| *Scrippsiella bicarinata* | Outgroup |  | KF751927 |
| *Scrippsiella* cf. *erinaceus* | Outgroup |  | JN982398 |
| *Scrippsiella hangoei* | Outgroup |  | EF205016 |
| *Scrippsiella* sp. | Outgroup | HQ845330 |  |
| *Scrippsiella sweeneyae* strain CCCM 280 | Outgroup | AF274276 |  |
| *Scrippsiella trochoidea* strain CCMP2271 | Outgroup | HM483396 | HM483396 |

**FIGURE S1.** Phylogenetic tree including LSU rDNA sequences of Pfiesteriaceae representatives. Outgroup sequences are represented by Thoracosphaeraceae representatives. Sequences generated in this study are in bold. Values in the nodes represent bootstrap statistical support (%) and Bayesian Posterior Probability. Only values >80% and >0.95 respectively are shown.

 **FIGURE S2.** Phylogenetic tree including SSU rDNA sequences of Pfiesteriaceae representatives. Outgroup sequences are represented by Thoracosphaeraceae representatives. Sequences generated in this study are in bold. Values in the nodes represent bootstrap statistical support (%) and Bayesian Posterior Probability. Only values >70% and >0.95 respectively are shown.
